# Fisetin Alleviates Inflammation and Oxidative Stress in Deep Vein Thrombosis via MAPK and NRF2 Signaling Pathway

**DOI:** 10.3390/ijms25073724

**Published:** 2024-03-27

**Authors:** Hao Liu, Qiulun Lu

**Affiliations:** Key Laboratory of Cardiovascular and Cerebrovascular Medicine, College of Pharmacy, Nanjing Medical University, 101 Longmian Avenue, Jiangning District, Nanjing 211166, China; haoliu@stu.njmu.edu.cn

**Keywords:** Fisetin, deep vein thrombosis, inflammation, oxidative stress, MAPK, NRF2

## Abstract

Oxidative stress and inflammation play pivotal roles in the progression of deep vein thrombosis (DVT). Fisetin has demonstrated promising pharmacological features; however, its underlying mechanisms in DVT remain elusive. In our study, we investigated the effects and underlying mechanisms of Fisetin on a DVT mouse model. The protective effects of Fisetin on DVT were evaluated by comparing the size of thrombosis and detecting the mRNA expression levels of pro-inflammatory cytokines. After that, the biological processes were studied via transcriptomics after Fisetin administration. The antioxidant effect was evaluated and explained via NRF2 signaling pathway. Finally, the anti-inflammatory effect was explained according to KEGG analysis and the final mechanism was verified via Western blot. Our results found that the mRNA expression levels of pro-inflammatory cytokines were inhibited by Fisetin. Moreover, transcriptomic studies suggested that MAPK signaling pathway may be associated with the anti-inflammatory activity of Fisetin. Then, we confirmed that Fisetin administration significantly inhibited the activation of typical pro-inflammatory signaling pathways via Western blot. Finally, the results of Western blot showed that Fisetin significantly activated NRF2 signaling pathway and induced the expression of downstream antioxidant enzymes. Our findings suggested that Fisetin exhibits potential therapeutic effects on DVT through its ability to attenuate inflammation and oxidative stress. The underlying mechanism may involve the suppression of MAPK-mediated inflammatory signaling pathway and activation of NRF2-mediated antioxidant signaling pathway.

## 1. Introduction

Deep vein thrombosis (DVT) refers to the abnormal coagulation of blood within the deep veins, leading to impaired blood flow in the venous lumen, resulting in local pain, swelling, and difficulty in movement. Although DVT primarily affects the deep veins of the lower extremities, it is not limited to the legs. DVT and its complications have become one of the leading causes of disability and death worldwide [1].

Unlike arterial thrombosis, the mechanism of DVT formation is still under exploration. While DVT has long been attributed to disorders of blood coagulation, recent research suggests that immune and inflammatory processes also play pivotal a role [2]. Numerous studies have reported that some traditional Chinese medicines possess excellent anti-coagulant and anti-thrombotic effects, suggesting their unique advantages and therapeutic efficacy in preventing and treating DVT [3]. Research has confirmed that traditional Chinese medicines can inhibit neutrophil activation, block neutrophil adhesion to endothelial cells, regulate inflammatory factors, and interrupt the inflammatory response through multiple targets, including inhibiting NF-κB activity [4].

Fisetin is a flavonoid polyphenol compound found in various fruits, vegetables, and medicinal plants such as *Rhus succedanea* and *Toxicodendron vernicifluum*. In recent years, Fisetin has been studied for its pharmacological activities widely, including anti-tumor, anti-inflammatory, antioxidant, and neuroprotective effects. Studies have indicated that Fisetin exerts its anti-inflammatory effects by inhibiting NF-κB activation, p38 MAPK phosphorylation, downregulating mast cell activity, and limiting interactions between mast cells and activated T cells [5]. Additionally, the NRF2-antioxidant response element (NRF2-ARE) pathway has been identified as one of the main pathways through which Fisetin exerts its antioxidant activity [6]. In past studies, NRF2/KEAP1 signaling was found to play a key role in several cancerous and non-cancerous diseases. Nuclear factor-erythroid 2-related factor 2 (NRF2) signaling can modulate many antioxidant enzymes impaired in preeclampsia (PE), reducing inflammation. Appropriate modulation of NRF2 signaling could ameliorate many of the placental dysfunctions, thereby improving pregnancy outcomes [7]. Moreover, NRF2 has been identified to have a dual function. On the one hand, it promotes angiogenesis and cancer cell metastasis while also leading to resistance to drugs and chemotherapy. On the other hand, NRF2 can increase glutathione expression and proliferation to protect cells from reactive oxygen species (ROS). In breast cancer cells, by reciprocally regulating each other, p53 and NRF2 play an important role in regulating cell survival and death pathways [8]. However, its role in alleviating thrombosis has not been discovered until now.

In a mouse model of deep vein thrombosis, we observed a significant increase in inflammatory factors, consistent with recent findings suggesting the important role of the inflammatory process in DVT formation. Given Fisetin’s potent anti-inflammatory and antioxidant properties, we attempted Fisetin treatment for deep vein thrombosis and achieved positive results. Furthermore, we elucidated the anti-inflammatory and antioxidant mechanisms of Fisetin in inhibiting thrombus formation, providing a new treatment approach for clinical DVT patients.

## 2. Results

### 2.1. Fisetin Protected against Venous Thrombosis following IVC Ligation

**Figure 1:**
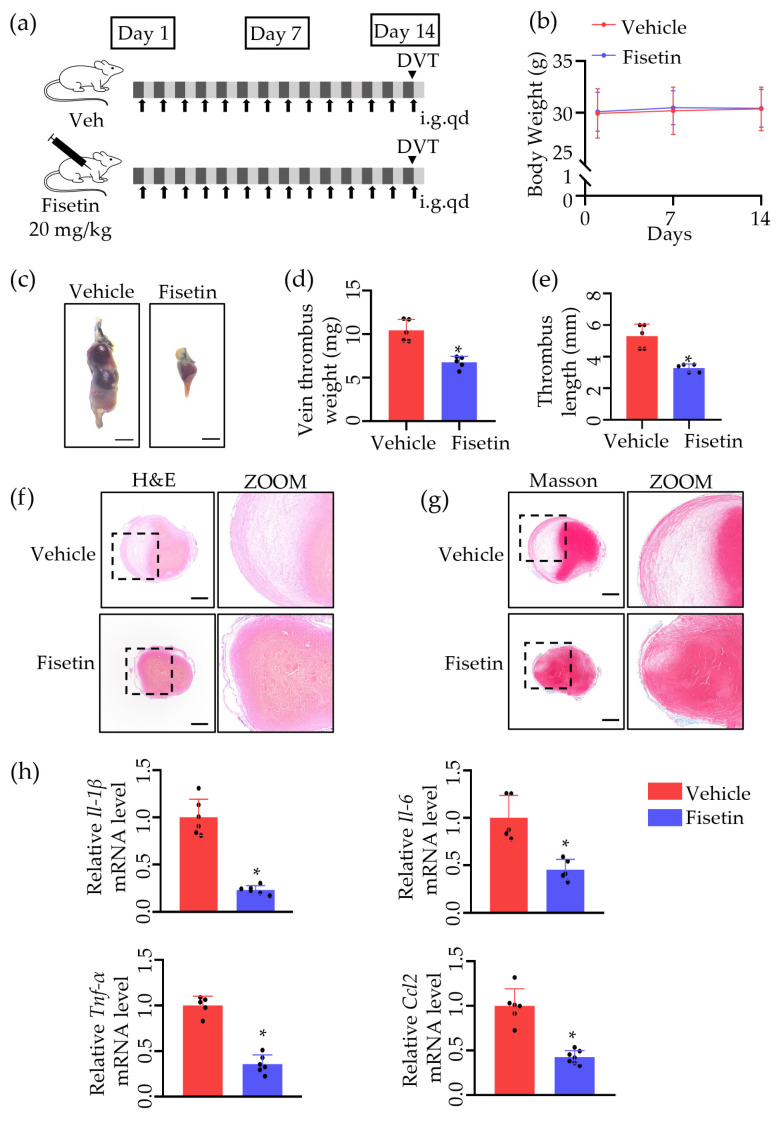
Fisetin protected against venous thrombosis following IVC ligation. (**a**) Schematic diagram of the experimental procedure. Veh, vehicle; i.g., intragastric administration; qd, once per day. Arrows represent daily intragastric administration, arrow heads represent the day of surgery. (**b**) The change of mice weight in 14 days (*n* = 6). (**c**) The thrombi induced via 24 h of inferior vena cava (IVC) ligation were harvested. Scale bar, 1 mm (*n* = 6). (**d**) The thrombi weights are indicated by dots in the graph (*n* = 5). (**e**) The thrombus length is indicated by dots in the graph (*n* = 5). (**f**) Representative image of H&E staining of thrombi. Scale bar, 300 μm (*n* = 5). (**g**) Representative image of Masson staining of thrombi. Scale bar, 300 μm (*n* = 5). (**h**) The mRNA levels of *Il-1β*, *Il-6*, *Mcp-1* and *TNFα* of different groups (*n* = 5). The dots represent the number of mice. All data are presented as means ± SD. * *p* < 0.05.

To investigate the role of Fisetin in DVT in vivo, we established a mouse DVT model by ligating the inferior vena cava (IVC). For 14 days before surgery, WT mice were treated with 20 mg/kg vehicle or Fisetin via intragastric administration consecutively, and thrombus tissues were collected 24 h post-surgery to evaluate thrombus formation (Figure 1a). Fisetin treatment did not significantly affect mouse body weight (Figure 1b) and effectively inhibited thrombus formation. Compared to the vehicle group, mice pre-treated with Fisetin showed a significant reduction in thrombus size (Figure 1c), with thrombus weight decreasing from 10.4 mg to 6.7 mg (Figure 1d). Similarly, thrombus length also significantly decreased from 5.3 mm to 3.3 mm (Figure 1e). Moreover, Fisetin reduced the infiltration of inflammatory cells into the thrombus (Figure 1f). However, there was no collagen deposition inside the thrombus in either group (Figure 1g), possibly due to the relatively short modeling time, consistent with previous reports [9]. The mRNA expression levels of pro-inflammatory cytokines such as *Il-1β*, *Il-6*, *Tnf-α*, and *Ccl2* were significantly inhibited by Fisetin (Figure 1h, Table 1). These results suggested that Fisetin pretreatment effectively inhibits thrombus formation.

### 2.2. Fisetin Prevented DVT without Heart, Liver, and Kidney Toxicity

**Figure 2:**
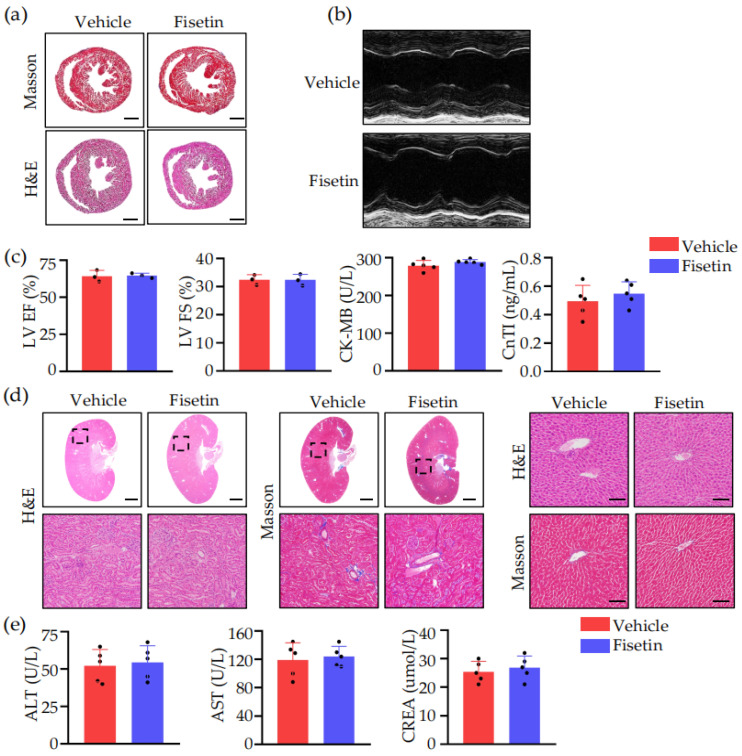
Fisetin prevented DVT without heart, liver, or kidney toxicity. (**a**) Representative image of H&E staining and Masson staining of heart of DVT mice treated with vehicle or Fisetin. Scale bar, 1 mm (*n* = 5). (**b**) Echocardiographic images comparing left ventricular function in DVT mice treated with vehicle or Fisetin (*n* = 3). (**c**) The left ventricular ejection fraction (LVEF), fractional shortening (LVFS), the level of CK-MB and CnTI were measured in DVT mice treated with vehicle or Fisetin (*n* = 3–5). (**d**) Representative images of H&E staining and Masson staining in the kidney and liver of DVT mice treated with vehicle or Fisetin. Scale bars, 1 mm (*n* = 5). (**e**) Serum ALT, AST, and CREA were measured. ALT, alanine transaminase; AST, aspartate aminotransferase; CREA, creatinine (*n* = 5). All data are presented as means ± SD.

Currently, its potential toxicity to the heart, liver, and kidneys remains unknown. Therefore, 24 h after surgery, we performed H&E and Masson staining on the hearts, livers, and kidneys of WT mice pretreated with Fisetin or vehicle, conducted ultrasound examination of mouse hearts, and tested their blood. It was found that Fisetin did not cause significant pathological changes in the hearts, livers, or kidneys, and there was no significant impact on index such as ejection fraction (EF), fractional shortening (FS), creatine kinase-MB (CK-MB), cardiac troponin I (CTnI), alanine aminotransferase (ALT), aspartate aminotransferase (AST), or creatinine (CREA) (Figure 2).

### 2.3. Fisetin Played a Role in Anti-Inflammation and Leukocytes Adhesion Inhibition in DVT Mice Model

**Figure 3:**
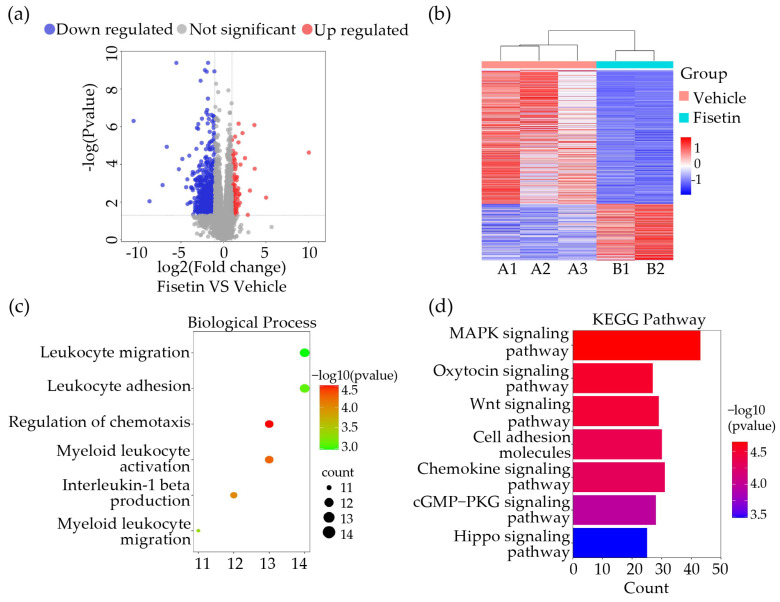
Fisetin played a role in anti-inflammation and leukocytes adhesion inhibition in DVT mice model. (**a**) Volcano plot of differential expression genes (DEGs) after treatment with 14-day Fisetin. Upregulated genes are indicated by red plots, downregulated genes by blue plots, and genes with no significant difference by gray plots (*n* = 2–3). (**b**) Heatmap for DEGs in Fisetin 14d vs. Veh. Three samples, each containing three mixed thrombi, were included in Veh group and two samples (three mixed thrombi) were included in Fisetin 14d group (*n* = 2–3). (**c**) Gene Ontology (GO) biological process (BP) analysis of DEGs. (**d**) KEGG enrichment analysis for DEGs identified via transcriptomics analysis.

To further investigate the mechanism by which Fisetin regulates thrombus formation, we performed RNA sequencing experiments to measure molecular changes in gene expression levels 24 h after IVC ligation in treated and untreated mouse groups. Setting *p* value < 0.05, log2FoldChange ≤ −0.58 and log2FoldChange ≥ 0.58, we found a total of 611 down-regulated differentially expressed genes (DEGs) and 114 up-regulated DEGs in thrombi of the Fisetin-treated group compared to the vehicle group (Figure 3a,b). Gene ontology (GO) biological pathway enrichment analysis of all DEGs between Fisetin and vehicle groups revealed that Fisetin appeared to affect leukocyte activation and adhesion (Figure 3c). Next, we conducted enrichment analysis of RNA-seq results using the Kyoto Encyclopedia of Genes and Genomes (KEGG) database to explore pathways involved in Fisetin’s role in thrombus formation, suggesting Fisetin might participate in the mitogen-activated protein kinase (MAPK) signaling pathway (Figure 3d). These results indicated that Fisetin is closely associated with the inflammatory response during thrombus formation in mice.

### 2.4. Fisetin Suppressed Leukocytes Accumulation in DVT Mice Model

**Figure 4:**
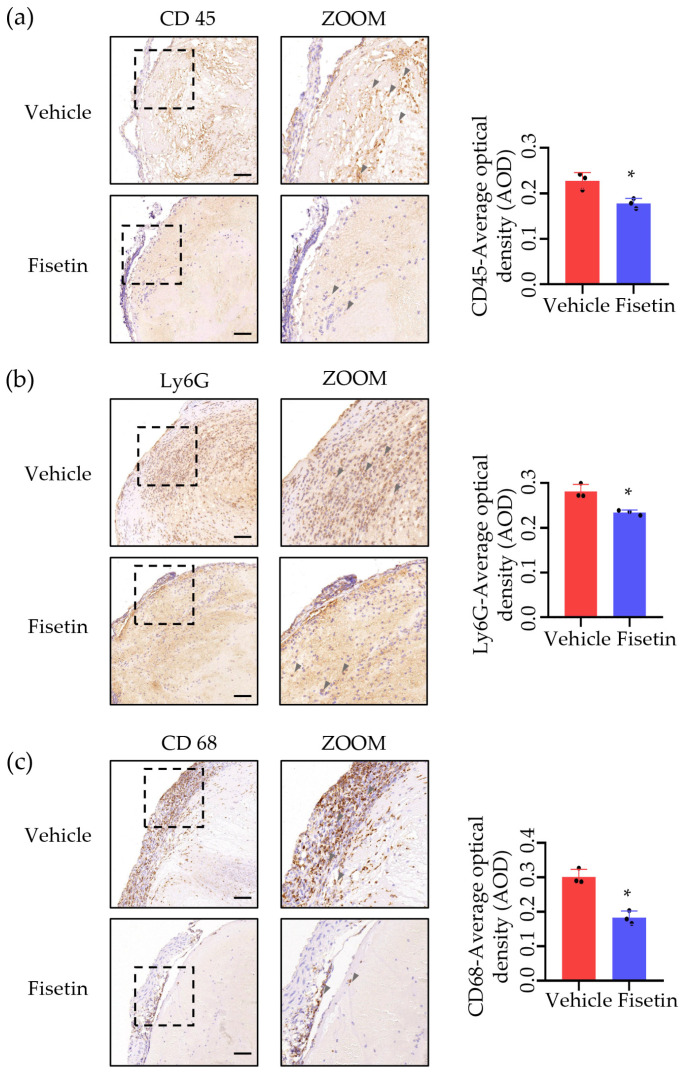
Fisetin suppressed leukocytes accumulation in DVT mice model. (**a**–**c**) Representative immunohistochemistry staining for CD45 (leukocyte marker), Ly6G (neutrophil marker), and CD68 (macrophages marker). Scale bar, 100 μm (*n* = 3). The positive cells in the thrombi were quantified and presented as average optical density in a thrombus. All data are presented as means ± SD. * *p* < 0.05.

Based on the above RNA-seq results, we investigated the distribution of various white blood cells within the thrombus. We performed immunohistochemical staining for CD68 (macrophage marker), Ly6G (neutrophil marker), and CD45 (leukocyte marker) on thrombi from vehicle and Fisetin-treated mice. Quantitative results showed a decrease in positive signals after treatment, with a 39% decrease in CD68-positive signals (Figure 4a), a 20% decrease in Ly6G-positive signals (Figure 4b), and a 22% decrease in CD45-positive signals (Figure 4c). These results indicated that Fisetin can affect the infiltration of white blood cells into the thrombus.

### 2.5. Fisetin Attenuated Leukocytes Migration and Adhesion

**Figure 5:**
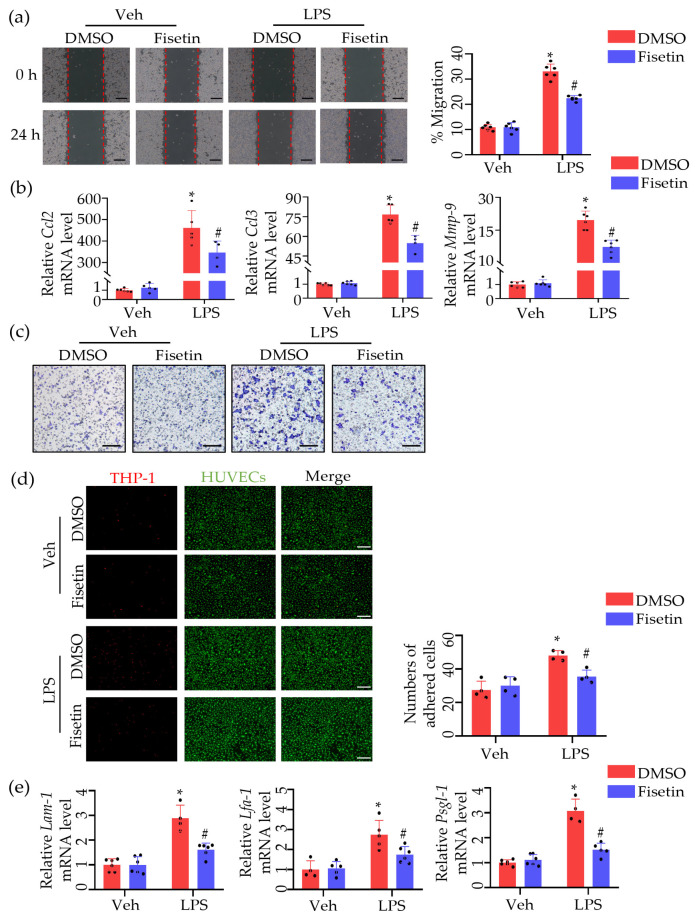
Fisetin attenuated leukocytes migration and adhesion. (**a**) Representative images of the scratch assay conducted on RAW264.7 cells. The top row shows images at time 0, and the bottom row shows images captured 24 h post-treatment. Quantitative analysis of the scratch wound healing assay after 24 hrs. Scale bar, 200 μm (*n* = 6). (**b**) The mRNA levels of *Ccl2*, *Ccl3* and *Mmp-9* of different groups. Representative images of cell migration in transwell assay of leukocyte under different conditions (*n* = 6). (**c**) Representative images of cell migration in transwell assay of leukocyte under different conditions. Scale bar, 150 μm (*n* = 3). (**d**) THP-1 cells adhesion to HUVECs were labeled via Dil and visualized. The histogram of the evaluation of adhered THP-1 cells assessed via cell count assay. Scale bar, 200 μm (*n* = 4). (**e**) The mRNA levels of *Lam-1*, *Lfa-1* and *Psgl*-1 of different groups (*n* = 6). All data are presented as means ± SD. Statistically significant difference analyzed via two-way ANOVA. * *p* < 0.05 vs. Veh + DMSO, # *p* < 0.05 vs. LPS + DMSO.

Next, to explore the effect of Fisetin on leukocyte adhesion and migration, we performed migration and adhesion studies using macrophage RAW264.7 cells and monocytes THP-1 cells, respectively. Scratch assay results showed that lipopolysaccharides (LPS) stimulations significantly promoted cell migration, which was significantly inhibited after Fisetin treatment (Figure 5a). Additionally, real-time qPCR analysis of migration markers (e.g., *Ccl2*, *Ccl3*, *Mmp-9*) mRNA expression levels revealed significant upregulation after LPS stimulation, which was significantly inhibited after Fisetin treatment (Figure 5b). Consistent with these results, transwell assay results further demonstrated that Fisetin inhibited leukocyte migration (Figure 5c). Experiments on THP-1 adhesion to human umbilical vein endothelial cells (HUVECs) showed that LPS stimulation significantly promoted THP-1 adhesion to HUVECs, which was significantly reduced after Fisetin treatment (Figure 5d). Similarly, changes in adhesion marker expression levels further confirmed these results (Figure 5e).

### 2.6. Fisetin Inhibited MAPK Signaling Pathway in DVT Mice Model

**Figure 6:**
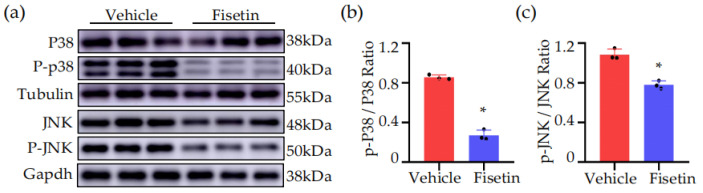
Fisetin inhibited MAPK signaling pathway in DVT mice model. (**a**) Representative Western blots for p-P38, P38, p-JNK, and JNK protein expressions in thrombi tissue extracts (*n* = 3). (**b**,**c**) The quantification of p-P38, P38, p-JNK, and JNK protein Western blots (*n* = 3). All data are presented as means ± SD. * *p* < 0.05.

All the phenotypes mentioned above demonstrated the potent anti-inflammatory function of Fisetin. The MAPK signaling pathway is widely recognized as a canonical pro-inflammatory signaling cascade and the KEGG enrichment analysis of DEGs suggested that the regulation of the MAPK signaling pathway might be the main route of action for Fisetin administration. We investigated the expressions of related proteins, including c-JunN-terminal kinase (JNK) and P38 signaling transduction. We found the significant increase in phosphorylated proteins of JNK and P38 in the model group. Fisetin treatment significantly decreased phosphorylation of JNK and P38 in DVT mice (Figure 6a), indicating that Fisetin can inhibit the MAPK signaling pathway in DVT mice (Figure 6b,c). In summary, these results suggested that the anti-inflammatory action of Fisetin may be associated with the inhibition of the MAPK signaling pathway.

### 2.7. Fisetin Reduced ROS in DVT Mouse Model

**Figure 7:**
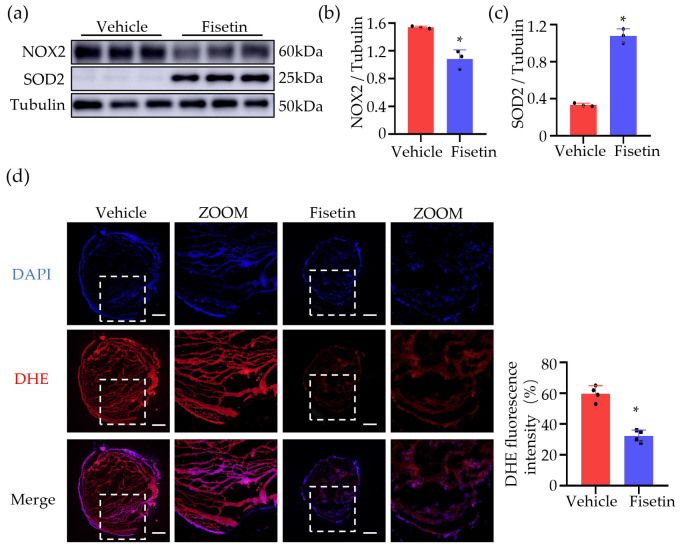
Fisetin reduced ROS in the DVT mouse model. (**a**) Representative Western blots for NOX2 and SOD2 protein expressions in thrombi tissue extracts (*n* = 3). (**b**,**c**) The quantification of NOX2 and SOD2 protein Western blots (*n* = 3). (**d**) Representative images of dihydroethidium (DHE) staining of thrombi. Scale bar, 250 μm (*n* = 4). The ROS production in the thrombus was quantified and presented as the mean fluorescence intensity. All data are presented as means ± SD. * *p* < 0.05.

ROS are associated with venous thrombosis and participate in regulating all major processes promoting venous thrombosis formation [10]. NOX2 (NADPH oxidase 2) is a pro-oxidant enzyme of phagocytes, and SOD2 (superoxide dismutase 2), which neutralizes toxic superoxide anion to form peroxide in mitochondria, is also an enzymatic antioxidant. To explore whether Fisetin can reduce the accumulation of ROS in DVT induced by venous stasis, we detected changes in the protein levels of NOX2 and SOD2 in the thrombus (Figure 7a–c). The results showed that compared to the model group, the Fisetin-treated group exhibited a significant decrease in NOX2 expression and a significant increase in SOD2 expression, indicating a significant antioxidant effect of Fisetin in thrombus formation. Then we stained the blood vessels, including the thrombus, with dihydroethidium (DHE) (Figure 7d). To quantify and measure ROS production, we measured fluorescence intensity in different groups and found that Fisetin pretreatment reduced ROS generation by 50% in the thrombus compared to the vehicle pretreatment group (Figure 7d). In conclusion, in the DVT mouse model, Fisetin effectively regulated venous thrombosis formation by reducing ROS accumulation.

### 2.8. Fisetin Activated NRF2-Mediated Antioxidant Pathway in DVT Mouse Model

**Figure 8:**
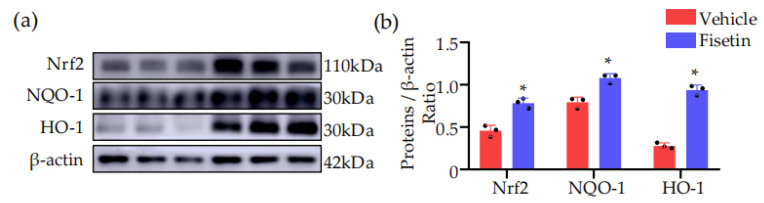
Fisetin activated NRF2-mediated antioxidant pathway in the DVT mouse model. (**a**) Representative Western blots for NRF2, HO-1 and NQO-1 protein expressions in thrombi tissue extracts (*n* = 3). (**b**) The quantification of NRF2, HO-1 and NQO-1 protein Western blots (*n* = 3). All data are presented as means ± SD. * *p* < 0.05.

After confirming the antioxidant effect of Fisetin, we further explored its potential mechanism. NRF2 plays a crucial role in the antioxidant system [11]. Under oxidative stress, activated NRF2 translocates to the nucleus, leading to the expression of enzymatic antioxidants, including heme oxygenase-1 (HO-1) and NAD(P)H quinone oxidoreductase-1 (NQO-1) [12]. The results showed that compared to the model group, the Fisetin-treated group significantly activated NRF2 signaling transduction and increased the expression level of HO-1 and NQO-1 (Figure 8a,b). These results suggested that the antioxidant effect of Fisetin associated with the activation of the NRF2 signaling pathway.

## 3. Discussion

The incidence of deep vein thrombosis (DVT) is gradually increasing and has become the third major threat to human health in peripheral vascular diseases [13]. DVT formation is often caused by factors such as trauma, surgery, pregnancy, and prolonged bed rest. Reports indicate that about 50% of deep vein thrombosis patients develop complications such as lower limb swelling, pigmentation, ulcers, etc., due to inadequate understanding and delayed treatment, seriously affecting patients’ lives and work [14]. Actively controlling DVT is of great research significance. In recent years, Fisetin has been widely studied for its pharmacological activities such as its anti-tumor, anti-inflammatory, antioxidant, and neuroprotective effects. In this study, we investigated the effects and underlying mechanisms of Fisetin on DVT. Our results indicated that Fisetin alleviated oxidative stress by activating the NRF2 signaling pathway and mitigated inflammation by inhibiting the MAPK signaling pathway.

Oxidative stress plays a crucial role in the pathogenesis and progression of cardiovascular diseases. Studies have found that oxidative stress reactions are closely related to thrombus formation. The body undergoes oxidative stress reactions due to worse antioxidant capacity and increased free radicals. Antioxidants in the normal body alleviate oxidative stress by scavenging enzymes such as superoxide dismutase (SOD) and glutathione peroxidase (GSH-Px), thereby reducing endothelial damage, maintaining vascular dilation function, and inhibiting thrombus formation [4]. It has also been shown that thrombus formation is the result of platelet activation and aggregation in circulation. NOX2 is a homologue of NADPH oxidase expressed in platelets and is an important regulatory factor in platelet activation-associated thrombus formation [15]. Mechanistically, Fisetin exerts its antioxidant effects mainly through activating NRF2, inducing HO-1 expression [16], inhibiting ROS generation, and regulating the level of the antioxidant enzyme glutathione [17]. The results of this study show that the expression levels of SOD2 were increased and the expression levels of NOX2 were decreased in the Fisetin-treated group. Meanwhile, the reactive oxygen levels in the thrombus were also decreased in DHE staining in the Fisetin-treated group. Fisetin treatment indeed significantly activated NRF2 signaling transduction and increased the expression of downstream proteins (HO-1 and NQO-1). In summary, these results suggested that Fisetin alleviates oxidative stress in the deep vein thrombosis mouse model by activating the NRF2 signaling pathway.

In addition to oxidative stress, inflammation is another key promoting factor in the pathogenesis of DVT. Evaluated research has shown that the activation of inflammatory signals and infiltration of inflammatory cells are crucial for the development of DVT [17]. Therefore, we also investigated the effects of Fisetin on inflammatory response and found that Fisetin significantly inhibited the expression of various inflammatory cells and pro-inflammatory cytokines. Subsequently, mRNA sequencing of thrombi was employed to elucidate the potential mechanisms of Fisetin’s anti-inflammatory action in DVT. We found that Fisetin’s anti-inflammatory effect may be attributed to its inhibition of the MAPK pathway. As a typical pro-inflammatory pathway, the overactivation of the MAPK pathway has been demonstrated to be closely associated with various inflammatory diseases. Previous studies have reported that Fisetin can exert anti-inflammatory effects by inhibiting the expression of inflammatory mediators such as Toll-like receptor 4, cyclooxygenase 2, prostaglandin E2, TNF-α, interleukins, and chemokines through pathways including NF-κB, PI3K-Akt, and MAPK [18].

As previously mentioned, there exists an intricate interplay between oxidative stress and inflammation. A similar association can be observed between the NRF2 and MAPK signaling pathways. Research has demonstrated that the inhibition of MAPK signaling transduction can augment NRF2 activity and HO-1 production [19]. The MAPK family has the ability to influence NRF2 activity, while conversely, NRF2 activity can regulate the activation of the MAPK signaling pathway [20]. Impairment in NRF2 function leads to the activation of MAPK signaling and a subsequent elevation in inflammation [21]. Consequently, aberrant NRF2 and MAPK signaling transduction jointly contribute to the development of DVT by regulating oxidative stress and inflammation. Although further elucidation is required regarding the intricate interplay between these mechanisms, the directionality of these factors in DVT is clear. Nevertheless, our research results demonstrated that Fisetin not only activates the NRF2 signaling pathway but also inhibits the MAPK signaling pathway, thereby synergistically alleviating oxidative stress and inflammation, thus preventing the formation of deep vein thrombosis.

This study validated the close association of oxidative stress and inflammation with the mechanism of deep vein thrombosis formation, and our findings introduced Fisetin as an effective agent for protecting against deep vein thrombosis. Furthermore, these findings provided compelling evidence that NRF2 and MAPK signaling pathways are promising targets for preventing deep vein thrombosis.

## 4. Materials and Methods

### 4.1. Reagents

Fisetin powder (Cat No. HY-N0182R) purchased from MCE (Monmouth Junction, NJ, USA). LPS (Cat No. SMB00704) and Dimethyl Sulfoxide (DMSO) (Cat No. D1435) were procured from Sigma-Aldrich (St. Louis, MO, USA), hematoxylin-eosin (Cat No. G1004), Masson staining kits (Cat No. G1006) and RPMI 1640 (Cat No. G4511) medium were provided by Servicebio (Wuhan, China). TRIzol Reagent (Cat No.15596018CN) was procured from Invitrogen (Waltham, MA, USA), the HiScript^®^ II Q RT SuperMix kit (Cat No. Q441-02) and the Power SYBR Green PCR Master Mix (Cat No. Q141-02) were obtained from Vazyme (Nanjing, China), dihydroethidium (Cat No. S0063) was procured from Beyotime (Shanghai, China), DMEM high-glucose medium (Cat No. BC-M-005) was provided by Bio-Channel (Nanjing, China). Antibodies against p-JNK (Cat No. a4668S) and p-p38 (Cat No. 4511S) were procured from Cell Signaling Technology (Danvers, MA, USA). Antibodies against HO-1 (Cat No. 10701-1-AP), c-Jun N-terminal kinase (JNK) (Cat No. 17572-1-AP), Nrf2 (Cat No. 16396-1-AP), NQO-1 (Cat No. 11451-1-AP), SOD2 (Cat No. 24127-1-AP), Gapdh (Cat No. 60004-1-Ig) and β-actin (Cat No. 66009-1-Ig) were obtained from Proteintech (Wuhan, China). CD68 (Cat No. ab283654), CD45 (Cat No. ab40763) and Ly6G (Cat No. ab238132) antibody were provided by Abcam (Cambridge, MA, USA).

### 4.2. Mice

All wild type mice (aged 8–13 weeks, 20–30 g) on the background of C57BL/6J were procured from the Animal Core Facility of Nanjing Medical University (Nanjing, China). Both sexes were included. All animals were kept in a temperature-controlled and specific pathogen-free (SPF) condition, with a 12:12 h dark-light cycle and provided with food and water ad libitum. All the experimental procedures performed on mice were approved by the Animal Care and Use Committee of Nanjing Medical University (IACUC-2212021).

### 4.3. Deep Vein Thrombosis (DVT) Surgery in Mice

The DVT mouse model was conducted as previously described [22]. Mice were anesthetized with 4% isoflurane mixture, followed by maintenance anesthesia with 2% isoflurane mixture. A laparotomy was performed along the ventral midline to expose the abdominal cavity in supine mice. A non-absorbable 7-0 suture was used to separate the inferior vena cava (IVC) from the aorta carefully and the IVC was ligated below the left renal vein. Additionally, all lateral branches and back branches were tied off using 7-0 stitches. A two-layer closure was applied to the laparotomy incision. The mice were euthanized after 24 h and perfused with 0.9% saline solution through their hearts. Thrombi weight was measured after the thrombus-containing IVC was excised from the ligated site (below the left renal vein) to the confluence of the common iliac vein.

### 4.4. Experimental Groups

To investigate the role of Fisetin on DVT, we divided all mice into two groups. Fisetin was first dissolved in 0.1% (*v*/*v*) DMSO and then diluted with physiological saline (0.9%) such that the total administered volume was in 0.9% physiological saline [23]. Mice were pre-treated with Fisetin (20 mg/kg/d) [23,24] for 7 days before DVT surgery and the vehicle groups were treated with 0.9% physiological saline for 7 days before DVT surgery. The Fisetin and vehicle were administered intragastrically (i.g.) to the mice.

### 4.5. Hematoxylin and Eosin (HE) and Masson Staining

IVC tissues containing thrombus, hearts, livers, and kidneys were fixed in 4% paraformaldehyde at 4 °C overnight and paraffin-embedded, respectively. For staining, the sample section slides were deparaffinized and hydrated via graded ethanol washes. Next, the sample section slides were stained with hematoxylin-eosin and Masson staining as previously described [25]. A light microscope was used for observation.

### 4.6. Cell Culture

THP-1 monocytes were maintained in RPMI 1640 medium which contained 10% fetal bovine serum (FBS). RAW264.7 cells were maintained in DMEM high-glucose medium supplemented with 10% FBS. Primary human umbilical vein endothelial cells (HUVECs) were isolated from the human umbilical vein (Shanghai Universal Biotech Co., Ltd., Shanghai, China) and maintained in endotoxin-free endothelial cell medium (ECM) containing 10% FBS, endothelial cell growth supplements and 1% penicillin-streptomycin. All the cells were incubated at 37 °C and 5% CO_2_ in a humidified culture incubator.

### 4.7. Quantitative Real-Time PCR (qRT-PCR)

The TRIzol Reagent was used to isolate total RNA from the transfected 293T cell. A HiScript^®^ II Q RT SuperMix kit was used for reverse transcription and the Power SYBR Green PCR Master Mix for reverse transcription PCR reaction with PCR primers. The gene expression levels were measured via the StepOne™ Real-Time PCR System.

### 4.8. Immunohistochemistry

The thrombi tissues were collected from the DVT mouse, and were fixed in 4% paraformaldehyde for 24 h, subsequently embedded in paraffin, and sectioned at 3 µm for staining. For CD68, Ly6G, and CD45 immunohistochemistry staining, thrombi sections were incubated overnight at 4 °C with the respective primary antibody, followed by secondary antibodies incubation for 1 h at room temperature. The average optical density (AOD) of CD45, Ly6G, and CD68 immunohistochemistry (IHC) staining intensity within the thrombus tissue was quantitatively measured via Image J 1.52V. Three areas of equal size were randomly selected for each tissue.

### 4.9. Western Blot

The cell lysates were obtained from thrombi tissues with RIPA buffer supplemented with protease inhibitors and phosSTOP phosphatase inhibitors. The protein concentrations were determined with bicinchoninic acid assay protein assay kits. Cell lysates (40 μg) were loaded and separated by SDS/PAGE. The protein bands were transferred to PVDF membrane then blocked with 10% milk at 4 °C for 1.5 h and incubated with antibody (1:2000) at 4 °C for 10 h. Then, secondary antibodies were used and incubated at room temperature for one hour. These results were visualized using ECL Western blotting substrate.

### 4.10. THP-1 Monocytes Adhesion Assays

To mimic the interactions between monocytes and endothelial cells in vivo, THP-1 monocytes and HUVECs were co-cultured in vitro to evaluate the attachment of monocytes to HUVECs under different conditions. At 2 h before LPS treatment, the THP-1 monocytes were pre-treated with Fisetin (10 μm) or vehicle. Subsequently, 100 ng/mL LPS was added to THP-1 suspension and incubated for 12 h in the treatment groups. HUVECs were also treated with LPS for 12 h in the treatment group. After 12 h, a stock solution of Dil (1000 μm) was diluted 1:100 with medium. Then, the THP-1 suspension was centrifuged to remove the supernatant, 2 mL of the prepared Dil solutions were added to the THP-1 cells, and incubated for 20 min at 37 °C. The Dil solution was then removed via centrifugation and new, pre-warmed medium was added. Meanwhile, HUVECs were washed twice by pre-warmed PBS. THP-1 monocytes (2 × 10^5^ cells) labeled with Dil were added to each well of HUVEC-seeded 6-well plates. After 1 h of incubation at 37 °C, cells were washed gently with pre-warmed PBS to remove any unattached THP-1 cells. A fluorescence microscope was used to quantify the Dil marked THP-1 cells adhered to the Calcein labeled HUVECs. Control groups received the same treatment but LPS treatment.

### 4.11. Transwell Migration Assays

Transwell chambers were used for the migration assay. To eliminate the influence of serum, the cells were cultured in serum-free DMEM high-glucose medium for 10 h. Next, 200 μL RAW 264.7 cell suspensions without serum were added into the upper chamber at a density of 8 × 10^4^/well while 600 μL DMEM high-glucose medium supplemented with 10% FBS was added into the lower chamber. A quantity of 10 μm Fisetin was added to the upper chambers and preincubated for 2 h prior to LPS addition. After 24 h, cells in the upper wells were removed with a cotton swab and RAW 264.7 cells on the bottom of the transwell membrane were fixed with 4% paraformaldehyde, and stained with 0.1% crystal violet. The migrated cells were counted in five random fields via bright field microscopy.

### 4.12. Dihydroethidium Staining

The sections of the frozen thrombi tissues from DVT mice were stained with dihydroethidium for 35 min. The fluorescence microscope was used to produce images. The ImageJ 1.54V was used to analyze the ROS levels by calculating fluorescence intensity.

### 4.13. Statistical Analysis

All data were presented as the mean ± standard deviation (SD). The “*n*” represents the number of mice. Statistical analysis was performed with GraphPad Prism 8. The normality of the data was checked via Shapiro-Wilk test. For comparisons between two samples, an unpaired Student’s *t*-test was used. While statistical analysis of more than two samples, a one-way ANOVA was used, followed by Tukey’s multiple comparison test. *p* < 0.05 was considered statistically significant.

## 5. Conclusions

In conclusion, this study investigated the potential effects and underlying mechanisms of Fisetin on DVT. Our findings demonstrated that Fisetin exhibits effective therapeutic potential against deep vein thrombosis (DVT) through its ability to attenuate oxidative stress and inflammation. Furthermore, the activation of the NRF2-mediated antioxidant signaling pathway and inhibition of the MAPK-mediated inflammatory signaling pathway are implicated as key mechanisms underlying its efficacy.

## Figures and Tables

**Table 1:** Oligonucleotide primer sequences used for reverse transcription–quantitative polymerase chain reaction.

Primer Name	Sequence (5′ to 3′)
Gapdh-F	CTGAACGGGAAGCTCACTG
Gapdh-R	CATACTTGGCAGGTTTCTCCAG
β-actin-F	CGGTTCCGATGCCCTGAGGCTCTT
β-actin-R	CGTCACACTTCATGATGGAATTGA
Tnf-α-F	ATCGGTCCCCAAAGGGATGA
Tnf-α-R	GGTGGTTTGCTACGACGTG
Il-1β-F	GAAATGCCACCTTTTGACAGTG
Il-1β-R	TGGATGCTCTCATCAGGACAG
Il-6-F	TCTATACCACTTCACAAGTCGGA
Il-6-R	GAATTGCCATTGCACAACTCTTT
Ccl2-F	GCATCCACGTGTTGGCTC
Ccl2-R	CTCCAGCCTACTCATTGGGATCA
Psgl-1-F	AGGAGATAAGATGGCTGGTGC
Psgl-1-R	GCTTTCTCGGCTTCATCTGC
Lam-1-F	CCTCTGTTACACAGCTTCTTGC
Lam-1-R	GTACCCCACATCACAGTTGC
Lfa-1-F	TGTCTCGAACGTGTGACCAG
Lfa-1-R	CGTTGCCCTTGATACATTCCTG
Ccl3-F	TCCCAGCCAGGTGTCATTTTCC
Ccl3-R	TCAGGCATTCAGTTCCAGGTCA
Mmp-9-F	TCCTGGCTCTCCTGGCTTTC
Mmp-9-R	TAGCGGTACAAGTATGCCTCTG

## Data Availability

The data supporting the findings of this study are available from the corresponding author upon reasonable request.
